# Triphen­yl[2-(triphenyl­phosphanium­yl)eth­yl]phosphanium bis­(periodate)

**DOI:** 10.1107/S1600536811008725

**Published:** 2011-03-12

**Authors:** Mostafa Gholizadeh, Farrokhzad Mohammadi Zonoz, Mehrdad Pourayoubi, Maliheh Ebrahimpour, Maryam Salehabadi

**Affiliations:** a Department of Chemistry, Ferdowsi University of Mashhad, Mashhad, 91779, Iran; b Department of Chemistry, Sabzevar Tarbiat Moallem University, Sabzevar, Iran

## Abstract

In title salt, C_38_H_34_P_2_^2+^·2IO_4_^−^, the P atoms of the dication and the I atoms of the periodate anions are each in a slightly distorted tetra­hedral environment. In the dication, the two –P(C_6_H_5_)_3_ groups adopt a *gauche* conformation with respect to each other. In the crystal, several C—H⋯O hydrogen bonds between the cations and anions lead to a two-dimensional arrangement along (101).

## Related literature

For the synthesis and structures of related compounds, see: Barkell *et al.* (2008 ▶); Rizzoli *et al.* (2010 ▶).
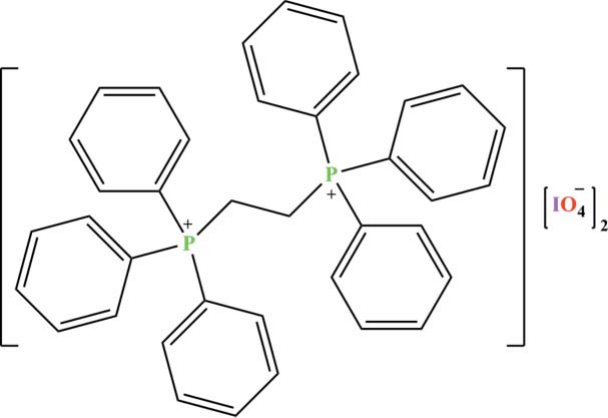


## Experimental

### 

#### Crystal data

C_38_H_34_P_2_^2+^·2IO_4_^−^
*M* *_r_* = 934.39
Monoclinic, 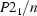

*a* = 9.2077 (18) Å
*b* = 18.387 (4) Å
*c* = 21.992 (4) Å
β = 94.37 (3)°
*V* = 3712.5 (13) Å^3^
*Z* = 4
Mo *K*α radiation
μ = 1.83 mm^−1^
*T* = 298 K
0.5 × 0.2 × 0.2 mm

#### Data collection

Stoe IPDS II diffractometer
Absorption correction: numerical [shape of crystal determined optically (*X-RED* and *X-SHAPE*; Stoe & Cie, 2005 ▶)] *T*_min_ = 0.649, *T*_max_ = 0.692
26606 measured reflections
9970 independent reflections
7229 reflections with *I* > 2σ(*I*)
*R*_int_ = 0.077

#### Refinement

*R*[*F* ^2^ > 2σ(*F* ^2^)] = 0.069
*wR*(*F* ^2^) = 0.190
*S* = 1.17
9970 reflections
451 parameters
H-atom parameters constrained
Δρ_max_ = 1.18 e Å^−3^
Δρ_min_ = −1.69 e Å^−3^

### 

Data collection: *X-AREA* (Stoe & Cie, 2005 ▶); cell refinement: *X-AREA* ▶); data reduction: *X-AREA*; program(s) used to solve structure: *SHELXS97* (Sheldrick, 2008 ▶); program(s) used to refine structure: *SHELXL97* (Sheldrick, 2008 ▶); molecular graphics: *ORTEP-3 for Windows* (Farrugia, 1997 ▶); software used to prepare material for publication: *WinGX* (Farrugia, 1999 ▶).

## Supplementary Material

Crystal structure: contains datablocks I, global. DOI: 10.1107/S1600536811008725/sj5109sup1.cif

Structure factors: contains datablocks I. DOI: 10.1107/S1600536811008725/sj5109Isup2.hkl

Additional supplementary materials:  crystallographic information; 3D view; checkCIF report

## Figures and Tables

**Table 1:** Hydrogen-bond geometry (Å, °)

*D*—H⋯*A*	*D*—H	H⋯*A*	*D*⋯*A*	*D*—H⋯*A*
C30—H30⋯O5^i^	0.93	2.43	3.351 (10)	172
C20—H20*B*⋯O8^ii^	0.97	2.38	3.338 (11)	170
C19—H19*B*⋯O4	0.97	2.44	3.397 (8)	171
C19—H19*A*⋯O7	0.97	2.30	3.135 (8)	144

Symmetry codes: (i) 
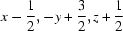
; (ii) 
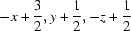
.

**Fig. 1.**
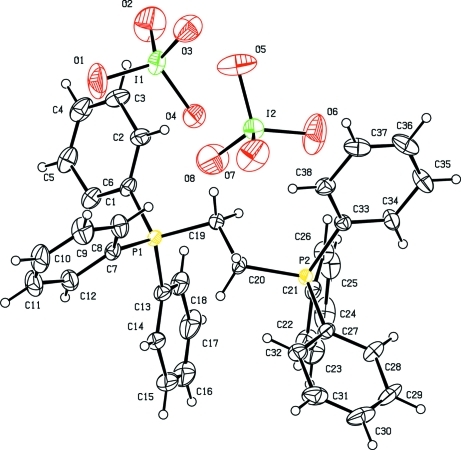
Molecular structure and atom labeling scheme for title salt with displacement ellipsoids drawn at the 50% probability level.

## supplementary crystallographic information

### Comment

In previous work, the syntheses and X-ray structures of some phosphonium salts, such as [C_6_H_5_–C_6_H_4_C(O)CH_2_P(C_6_H_5_)_3_]^+^[CF_3_SO_3_]^-^ (Rizzoli *et al.*, 2010) and [(C_6_H_5_)_3_PCH_2_C_6_H_2_(OCH_3_)_3_]^+^Cl^-^H_2_O (Barkell *et al.*, 2008) have been investigated. We report here the synthesis and crystal structure of a new phosphonium salt, [P(C_6_H_5_)_3_CH_2_CH_2_P(C_6_H_5_)_3_]^2+^, 2[IO_4_]^-^ (Fig. 1). In the cation of the title salt, the phosphorus atoms are found in a slightly distorted tetrahedral environment; the bond angles around the P atoms are in the range of 106.6 (3)° to 112.0 (3)° for P1 and 107.9 (3)° to 110.4 (3)° for P2. The two P(C_6_H_5_)_3_ groups are *gauche* to each other, the torsion angle P1—C19—C20—P2 is 136.1 (3)°. The I atoms of the two symmetrically independent periodate anions (labeled with I1 and I2) also display slightly distorted tetrahedral environments; for example, the bond angles around I1 are in the range of 108.0 (3)° to 111.2 (4)°. Several C—H···O hydrogen bonds (C···O distances are in the range of 3.135 (8) Å to 3.397 (8) Å) between the cations and anions lead to a two-dimensional arrangement along the (101) plane.

### Experimental

Preparation of bis (triphenylphosphonium) 1,2-ethane bromide: 1,2-Dibromoethane (15 mmol) was added to triphenylphosphine (30 mmol) and refluxed. After 5 h, the precipitate was filtered and washed with diethyl ether and dried.

Preparation of title salt: To a solution of bis (triphenylphosphonium) 1,2-ethane bromide (10 mmol) in H_2_O (25 ml), a solution of NaIO_4_ (20 mmol) in H_2_O (25 ml) was added and stirred. After 24 h, the precipitate was filtered and washed with H_2_O and crystallized from CH_3_CN at room temperature.

### Refinement

Carbon-bound H-atoms were placed in calculated positions, C—H = 0.93 Å (aromatic) and 0.97 Å (CH_2_), and were included in the refinement using a riding model approximation, with  U_iso_ = 1.2U_eq_ (C).

### Crystal data

**Table:**

C_38_H_34_P_2_^2+^·2IO_4_^−^	*F*(000) = 1848
*M**_r_* = 934.39	*D*_x_ = 1.672 Mg m^−^^3^
Monoclinic, *P*2_1_/*n*	Mo *K*α radiation, λ = 0.71073 Å
Hall symbol: -P 2yn	Cell parameters from 9970 reflections
*a* = 9.2077 (18) Å	θ = 2.2–29.3°
*b* = 18.387 (4) Å	µ = 1.83 mm^−^^1^
*c* = 21.992 (4) Å	*T* = 298 K
β = 94.37 (3)°	Plate, colorless
*V* = 3712.5 (13) Å^3^	0.5 × 0.2 × 0.2 mm
*Z* = 4	

### Data collection

**Table:**

Stoe IPDS II diffractometer	9970 independent reflections
Radiation source: fine-focus sealed tube	7229 reflections with *I* > 2σ(*I*)
graphite	*R*_int_ = 0.077
Detector resolution: 0.15 pixels mm^-1^	θ_max_ = 29.3°, θ_min_ = 2.2°
rotation method scans	*h* = −11→12
Absorption correction: numerical [shape of crystal determined optically (*X-RED* and *X-SHAPE*; Stoe & Cie, 2005)]	*k* = −24→25
*T*_min_ = 0.649, *T*_max_ = 0.692	*l* = −30→30
26606 measured reflections	

### Refinement

**Table:**

Refinement on *F*^2^	Primary atom site location: structure-invariant direct methods
Least-squares matrix: full	Secondary atom site location: difference Fourier map
*R*[*F*^2^ > 2σ(*F*^2^)] = 0.069	Hydrogen site location: inferred from neighbouring sites
*wR*(*F*^2^) = 0.190	H-atom parameters constrained
*S* = 1.17	*w* = 1/[σ^2^(*F*_o_^2^) + (0.0878*P*)^2^ + 4.1315*P*] where *P* = (*F*_o_^2^ + 2*F*_c_^2^)/3
9970 reflections	(Δ/σ)_max_ < 0.001
451 parameters	Δρ_max_ = 1.18 e Å^−^^3^
0 restraints	Δρ_min_ = −1.68 e Å^−^^3^

### Special details

**Table:**

Geometry. All e.s.d.'s (except the e.s.d. in the dihedral angle between two l.s. planes) are estimated using the full covariance matrix. The cell e.s.d.'s are taken into account individually in the estimation of e.s.d.'s in distances, angles and torsion angles; correlations between e.s.d.'s in cell parameters are only used when they are defined by crystal symmetry. An approximate (isotropic) treatment of cell e.s.d.'s is used for estimating e.s.d.'s involving l.s. planes.
Refinement. Refinement of *F*^2^ against ALL reflections. The weighted *R*-factor *wR* and goodness of fit *S* are based on *F*^2^, conventional *R*-factors *R* are based on *F*, with *F* set to zero for negative *F*^2^. The threshold expression of *F*^2^ > σ(*F*^2^) is used only for calculating *R*-factors(gt) *etc*. and is not relevant to the choice of reflections for refinement. *R*-factors based on *F*^2^ are statistically about twice as large as those based on *F*, and *R*- factors based on ALL data will be even larger.

### Fractional atomic coordinates and isotropic or equivalent isotropic displacement parameters (Å^2^)

**Table:**

	*x*	*y*	*z*	*U*_iso_*/*U*_eq_	
C17	0.5169 (13)	0.5057 (5)	0.3529 (4)	0.080 (3)	
H17	0.5649	0.4623	0.3626	0.096*	
I1	0.78527 (5)	0.81354 (3)	0.04912 (2)	0.05251 (15)	
I2	0.99789 (5)	0.50851 (2)	0.22398 (2)	0.04750 (13)	
P2	0.79094 (14)	0.76230 (7)	0.37105 (5)	0.0281 (3)	
P1	0.52902 (15)	0.66259 (7)	0.22621 (5)	0.0288 (3)	
C13	0.4736 (7)	0.6095 (3)	0.2891 (2)	0.0387 (12)	
C22	0.7211 (9)	0.6477 (4)	0.4439 (3)	0.0553 (18)	
H22	0.6486	0.6780	0.4568	0.066*	
C7	0.3896 (6)	0.7236 (3)	0.1975 (2)	0.0351 (11)	
C27	0.7309 (7)	0.8181 (3)	0.4310 (2)	0.0382 (12)	
C1	0.5723 (7)	0.6036 (3)	0.1652 (2)	0.0355 (11)	
C28	0.8096 (9)	0.8135 (4)	0.4882 (3)	0.0519 (16)	
H28	0.8910	0.7837	0.4941	0.062*	
C19	0.6887 (6)	0.7135 (3)	0.2547 (2)	0.0332 (10)	
H19A	0.7654	0.6800	0.2687	0.040*	
H19B	0.7236	0.7425	0.2219	0.040*	
C33	0.9613 (6)	0.7949 (3)	0.3475 (2)	0.0327 (10)	
C20	0.6531 (6)	0.7637 (3)	0.3075 (2)	0.0336 (11)	
H20A	0.5606	0.7491	0.3220	0.040*	
H20B	0.6425	0.8131	0.2924	0.040*	
C21	0.8146 (7)	0.6714 (3)	0.4001 (2)	0.0357 (11)	
C12	0.2447 (7)	0.7006 (4)	0.1924 (3)	0.0479 (14)	
H12	0.2183	0.6568	0.2096	0.058*	
C6	0.4850 (8)	0.5428 (3)	0.1532 (3)	0.0479 (15)	
H6	0.4116	0.5317	0.1786	0.057*	
C26	0.9184 (8)	0.6259 (3)	0.3797 (3)	0.0459 (14)	
H26	0.9783	0.6416	0.3502	0.055*	
C32	0.6125 (7)	0.8641 (3)	0.4224 (3)	0.0434 (13)	
H32	0.5630	0.8680	0.3841	0.052*	
C8	0.4276 (8)	0.7889 (4)	0.1725 (3)	0.0482 (14)	
H8	0.5239	0.8047	0.1759	0.058*	
C30	0.6429 (12)	0.8995 (4)	0.5272 (3)	0.067 (2)	
H30	0.6130	0.9265	0.5598	0.081*	
C31	0.5667 (10)	0.9049 (4)	0.4710 (4)	0.063 (2)	
H31	0.4860	0.9352	0.4655	0.075*	
C9	0.3191 (10)	0.8310 (5)	0.1422 (4)	0.066 (2)	
H9	0.3432	0.8755	0.1256	0.079*	
C4	0.6197 (10)	0.5157 (4)	0.0671 (3)	0.064 (2)	
H4	0.6365	0.4856	0.0344	0.077*	
C29	0.7614 (11)	0.8552 (5)	0.5358 (3)	0.067 (2)	
H29	0.8113	0.8527	0.5742	0.080*	
C2	0.6809 (8)	0.6204 (4)	0.1274 (3)	0.0491 (15)	
H2	0.7373	0.6619	0.1349	0.059*	
C5	0.5067 (10)	0.4989 (4)	0.1040 (3)	0.061 (2)	
H5	0.4473	0.4587	0.0953	0.073*	
C11	0.1412 (8)	0.7438 (5)	0.1615 (4)	0.064 (2)	
H11	0.0445	0.7289	0.1579	0.077*	
C23	0.7404 (12)	0.5777 (5)	0.4673 (4)	0.075 (3)	
H23	0.6809	0.5611	0.4967	0.091*	
C10	0.1786 (9)	0.8080 (5)	0.1363 (4)	0.069 (2)	
H10	0.1079	0.8362	0.1150	0.082*	
C3	0.7065 (10)	0.5760 (5)	0.0784 (3)	0.069 (2)	
H3	0.7810	0.5867	0.0536	0.083*	
C25	0.9331 (10)	0.5565 (4)	0.4036 (4)	0.064 (2)	
H25	1.0028	0.5252	0.3898	0.076*	
C24	0.8452 (12)	0.5334 (5)	0.4477 (5)	0.079 (3)	
H24	0.8577	0.4870	0.4642	0.094*	
C34	1.0851 (7)	0.7827 (4)	0.3851 (3)	0.0451 (14)	
H34	1.0800	0.7545	0.4200	0.054*	
C38	0.9676 (8)	0.8376 (3)	0.2951 (3)	0.0428 (13)	
H38	0.8840	0.8467	0.2698	0.051*	
O4	0.7872 (7)	0.8035 (4)	0.1293 (3)	0.0770 (18)	
C14	0.3675 (8)	0.6350 (5)	0.3252 (3)	0.0519 (16)	
H14	0.3179	0.6780	0.3155	0.062*	
O1	0.6096 (8)	0.8275 (6)	0.0168 (4)	0.117 (3)	
O3	0.8942 (9)	0.8885 (4)	0.0339 (3)	0.092 (2)	
C37	1.1017 (9)	0.8662 (4)	0.2815 (3)	0.0557 (18)	
H37	1.1079	0.8947	0.2468	0.067*	
C18	0.5491 (9)	0.5449 (4)	0.3023 (3)	0.0509 (16)	
H18	0.6196	0.5286	0.2774	0.061*	
C15	0.3371 (10)	0.5946 (7)	0.3765 (3)	0.077 (3)	
H15	0.2665	0.6107	0.4014	0.092*	
O7	0.9244 (10)	0.5937 (4)	0.2375 (4)	0.106 (3)	
C35	1.2175 (8)	0.8120 (4)	0.3713 (4)	0.0588 (19)	
H35	1.3007	0.8044	0.3973	0.071*	
O2	0.8591 (11)	0.7359 (5)	0.0176 (4)	0.120 (3)	
O5	1.0677 (11)	0.5069 (4)	0.1517 (4)	0.112 (3)	
O6	1.1384 (11)	0.4915 (6)	0.2794 (5)	0.132 (4)	
C16	0.4092 (12)	0.5326 (6)	0.3902 (4)	0.083 (3)	
H16	0.3883	0.5066	0.4247	0.100*	
C36	1.2246 (9)	0.8524 (4)	0.3193 (4)	0.067 (2)	
H36	1.3139	0.8707	0.3093	0.080*	
O8	0.8702 (12)	0.4403 (5)	0.2268 (4)	0.130 (4)	

### Atomic displacement parameters (Å^2^)

**Table:**

	*U*^11^	*U*^22^	*U*^33^	*U*^12^	*U*^13^	*U*^23^
C17	0.109 (8)	0.077 (6)	0.050 (4)	−0.039 (5)	−0.024 (5)	0.033 (4)
I1	0.0562 (3)	0.0506 (3)	0.0483 (2)	0.00431 (19)	−0.01228 (18)	−0.00348 (17)
I2	0.0502 (3)	0.0379 (2)	0.0542 (2)	0.00568 (17)	0.00268 (17)	−0.00459 (16)
P2	0.0279 (6)	0.0320 (6)	0.0239 (5)	−0.0021 (5)	−0.0013 (4)	0.0009 (4)
P1	0.0318 (7)	0.0309 (6)	0.0232 (5)	0.0021 (5)	−0.0005 (4)	−0.0021 (4)
C13	0.046 (3)	0.042 (3)	0.029 (2)	−0.007 (2)	0.003 (2)	0.003 (2)
C22	0.072 (5)	0.049 (4)	0.045 (3)	−0.017 (3)	0.002 (3)	0.010 (3)
C7	0.037 (3)	0.036 (3)	0.031 (2)	0.008 (2)	−0.004 (2)	−0.002 (2)
C27	0.044 (3)	0.045 (3)	0.027 (2)	−0.010 (2)	0.005 (2)	−0.005 (2)
C1	0.044 (3)	0.032 (3)	0.029 (2)	0.009 (2)	−0.004 (2)	−0.0062 (19)
C28	0.069 (5)	0.059 (4)	0.028 (3)	0.000 (3)	0.000 (3)	−0.005 (2)
C19	0.030 (3)	0.039 (3)	0.030 (2)	0.001 (2)	0.0000 (18)	−0.004 (2)
C33	0.033 (3)	0.031 (2)	0.034 (2)	−0.004 (2)	0.005 (2)	0.0018 (19)
C20	0.036 (3)	0.038 (3)	0.026 (2)	−0.001 (2)	−0.0031 (19)	−0.0090 (19)
C21	0.042 (3)	0.034 (3)	0.030 (2)	−0.005 (2)	−0.004 (2)	0.005 (2)
C12	0.037 (3)	0.054 (4)	0.052 (3)	0.003 (3)	−0.003 (3)	−0.005 (3)
C6	0.064 (4)	0.040 (3)	0.039 (3)	0.002 (3)	−0.002 (3)	−0.006 (2)
C26	0.052 (4)	0.033 (3)	0.053 (3)	0.001 (3)	−0.003 (3)	0.004 (2)
C32	0.045 (3)	0.045 (3)	0.041 (3)	0.001 (3)	0.008 (2)	−0.008 (2)
C8	0.044 (3)	0.042 (3)	0.057 (4)	0.001 (3)	−0.010 (3)	0.009 (3)
C30	0.108 (7)	0.054 (4)	0.044 (4)	−0.011 (5)	0.032 (4)	−0.014 (3)
C31	0.069 (5)	0.059 (4)	0.064 (4)	−0.002 (4)	0.029 (4)	−0.020 (4)
C9	0.064 (5)	0.056 (4)	0.074 (5)	0.015 (4)	−0.014 (4)	0.024 (4)
C4	0.083 (6)	0.059 (4)	0.049 (4)	0.026 (4)	−0.004 (4)	−0.020 (3)
C29	0.112 (7)	0.068 (5)	0.021 (3)	−0.025 (5)	0.006 (3)	−0.005 (3)
C2	0.051 (4)	0.057 (4)	0.040 (3)	−0.004 (3)	0.012 (3)	−0.011 (3)
C5	0.082 (6)	0.048 (4)	0.049 (4)	0.003 (4)	−0.014 (4)	−0.013 (3)
C11	0.038 (4)	0.074 (5)	0.078 (5)	0.015 (4)	−0.014 (3)	−0.018 (4)
C23	0.094 (7)	0.077 (6)	0.054 (4)	−0.038 (5)	−0.007 (4)	0.031 (4)
C10	0.055 (5)	0.073 (5)	0.073 (5)	0.028 (4)	−0.026 (4)	−0.004 (4)
C3	0.074 (6)	0.090 (6)	0.046 (4)	0.001 (5)	0.021 (4)	−0.025 (4)
C25	0.067 (5)	0.037 (3)	0.083 (5)	0.000 (3)	−0.016 (4)	0.008 (3)
C24	0.098 (7)	0.044 (4)	0.087 (6)	−0.024 (5)	−0.038 (5)	0.027 (4)
C34	0.031 (3)	0.052 (4)	0.051 (3)	−0.005 (3)	−0.004 (2)	0.008 (3)
C38	0.052 (4)	0.042 (3)	0.036 (3)	−0.006 (3)	0.009 (2)	0.008 (2)
O4	0.067 (4)	0.106 (5)	0.057 (3)	−0.007 (3)	−0.001 (3)	0.016 (3)
C14	0.047 (4)	0.074 (5)	0.034 (3)	−0.014 (3)	0.005 (2)	0.000 (3)
O1	0.068 (5)	0.158 (8)	0.117 (6)	0.024 (5)	−0.047 (4)	−0.020 (6)
O3	0.124 (6)	0.079 (4)	0.074 (4)	−0.027 (4)	0.012 (4)	0.006 (3)
C37	0.069 (5)	0.038 (3)	0.064 (4)	−0.005 (3)	0.029 (4)	0.010 (3)
C18	0.062 (4)	0.045 (3)	0.043 (3)	−0.005 (3)	−0.011 (3)	0.009 (3)
C15	0.068 (5)	0.128 (9)	0.037 (3)	−0.034 (6)	0.011 (3)	0.007 (4)
O7	0.136 (7)	0.074 (4)	0.107 (5)	0.062 (5)	0.003 (5)	−0.027 (4)
C35	0.033 (3)	0.056 (4)	0.087 (5)	−0.007 (3)	0.002 (3)	0.011 (4)
O2	0.138 (8)	0.085 (5)	0.135 (7)	0.039 (5)	−0.003 (6)	−0.040 (5)
O5	0.161 (9)	0.098 (6)	0.085 (5)	0.021 (5)	0.067 (6)	0.018 (4)
O6	0.121 (7)	0.136 (8)	0.128 (7)	0.052 (6)	−0.066 (6)	−0.025 (6)
C16	0.092 (7)	0.109 (8)	0.047 (4)	−0.044 (6)	−0.006 (4)	0.037 (5)
C36	0.050 (4)	0.055 (4)	0.099 (6)	−0.015 (4)	0.031 (4)	0.004 (4)
O8	0.155 (9)	0.120 (7)	0.118 (6)	−0.085 (7)	0.032 (6)	−0.020 (5)

### Geometric parameters (Å, °)

**Table:**

C17—C18	1.376 (10)	C26—C25	1.383 (9)
C17—C16	1.423 (16)	C26—H26	0.9300
C17—H17	0.9300	C32—C31	1.396 (8)
I1—O1	1.736 (7)	C32—H32	0.9300
I1—O2	1.747 (7)	C8—C9	1.393 (9)
I1—O3	1.752 (7)	C8—H8	0.9300
I1—O4	1.772 (6)	C30—C29	1.363 (13)
I2—O8	1.723 (8)	C30—C31	1.378 (13)
I2—O6	1.737 (8)	C30—H30	0.9300
I2—O7	1.741 (6)	C31—H31	0.9300
I2—O5	1.760 (6)	C9—C10	1.357 (13)
P2—C27	1.791 (6)	C9—H9	0.9300
P2—C33	1.792 (5)	C4—C3	1.377 (13)
P2—C21	1.796 (6)	C4—C5	1.402 (13)
P2—C20	1.814 (5)	C4—H4	0.9300
P1—C7	1.784 (5)	C29—H29	0.9300
P1—C1	1.793 (5)	C2—C3	1.386 (9)
P1—C13	1.798 (6)	C2—H2	0.9300
P1—C19	1.814 (6)	C5—H5	0.9300
C13—C14	1.387 (9)	C11—C10	1.359 (13)
C13—C18	1.396 (9)	C11—H11	0.9300
C22—C23	1.393 (11)	C23—C24	1.358 (15)
C22—C21	1.408 (9)	C23—H23	0.9300
C22—H22	0.9300	C10—H10	0.9300
C7—C8	1.377 (9)	C3—H3	0.9300
C7—C12	1.396 (9)	C25—C24	1.376 (14)
C27—C32	1.381 (9)	C25—H25	0.9300
C27—C28	1.406 (8)	C24—H24	0.9300
C1—C2	1.384 (9)	C34—C35	1.388 (9)
C1—C6	1.391 (9)	C34—H34	0.9300
C28—C29	1.398 (10)	C38—C37	1.395 (10)
C28—H28	0.9300	C38—H38	0.9300
C19—C20	1.539 (7)	C14—C15	1.398 (10)
C19—H19A	0.9700	C14—H14	0.9300
C19—H19B	0.9700	C37—C36	1.376 (12)
C33—C34	1.375 (8)	C37—H37	0.9300
C33—C38	1.398 (7)	C18—H18	0.9300
C20—H20A	0.9700	C15—C16	1.341 (16)
C20—H20B	0.9700	C15—H15	0.9300
C21—C26	1.370 (9)	C35—C36	1.370 (11)
C12—C11	1.379 (10)	C35—H35	0.9300
C12—H12	0.9300	C16—H16	0.9300
C6—C5	1.378 (9)	C36—H36	0.9300
C6—H6	0.9300		
			
C18—C17—C16	119.2 (9)	C27—C32—C31	120.4 (6)
C18—C17—H17	120.4	C27—C32—H32	119.8
C16—C17—H17	120.4	C31—C32—H32	119.8
O1—I1—O2	109.7 (4)	C7—C8—C9	118.7 (7)
O1—I1—O3	109.5 (5)	C7—C8—H8	120.7
O2—I1—O3	108.7 (5)	C9—C8—H8	120.7
O1—I1—O4	111.2 (4)	C29—C30—C31	121.0 (7)
O2—I1—O4	109.6 (4)	C29—C30—H30	119.5
O3—I1—O4	108.0 (3)	C31—C30—H30	119.5
O8—I2—O6	108.5 (6)	C30—C31—C32	119.0 (8)
O8—I2—O7	111.9 (5)	C30—C31—H31	120.5
O6—I2—O7	108.7 (4)	C32—C31—H31	120.5
O8—I2—O5	108.5 (4)	C10—C9—C8	121.1 (8)
O6—I2—O5	109.1 (5)	C10—C9—H9	119.5
O7—I2—O5	110.1 (4)	C8—C9—H9	119.5
C27—P2—C33	110.3 (3)	C3—C4—C5	121.3 (6)
C27—P2—C21	107.9 (3)	C3—C4—H4	119.4
C33—P2—C21	109.2 (3)	C5—C4—H4	119.4
C27—P2—C20	108.8 (3)	C30—C29—C28	121.3 (7)
C33—P2—C20	110.4 (3)	C30—C29—H29	119.3
C21—P2—C20	110.2 (3)	C28—C29—H29	119.3
C7—P1—C1	108.1 (2)	C1—C2—C3	120.6 (7)
C7—P1—C13	112.0 (3)	C1—C2—H2	119.7
C1—P1—C13	109.8 (3)	C3—C2—H2	119.7
C7—P1—C19	109.9 (3)	C6—C5—C4	119.0 (7)
C1—P1—C19	110.5 (3)	C6—C5—H5	120.5
C13—P1—C19	106.6 (3)	C4—C5—H5	120.5
C14—C13—C18	122.0 (6)	C10—C11—C12	121.0 (8)
C14—C13—P1	120.7 (5)	C10—C11—H11	119.5
C18—C13—P1	117.1 (5)	C12—C11—H11	119.5
C23—C22—C21	118.1 (8)	C24—C23—C22	120.7 (8)
C23—C22—H22	120.9	C24—C23—H23	119.7
C21—C22—H22	120.9	C22—C23—H23	119.7
C8—C7—C12	120.2 (6)	C9—C10—C11	120.0 (7)
C8—C7—P1	119.5 (5)	C9—C10—H10	120.0
C12—C7—P1	119.7 (5)	C11—C10—H10	120.0
C32—C27—C28	120.4 (6)	C4—C3—C2	118.9 (7)
C32—C27—P2	122.4 (4)	C4—C3—H3	120.5
C28—C27—P2	117.2 (5)	C2—C3—H3	120.5
C2—C1—C6	119.9 (5)	C24—C25—C26	120.4 (9)
C2—C1—P1	121.8 (5)	C24—C25—H25	119.8
C6—C1—P1	118.0 (5)	C26—C25—H25	119.8
C29—C28—C27	117.8 (7)	C23—C24—C25	120.6 (7)
C29—C28—H28	121.1	C23—C24—H24	119.7
C27—C28—H28	121.1	C25—C24—H24	119.7
C20—C19—P1	110.9 (4)	C33—C34—C35	120.5 (6)
C20—C19—H19A	109.5	C33—C34—H34	119.8
P1—C19—H19A	109.5	C35—C34—H34	119.8
C20—C19—H19B	109.5	C37—C38—C33	118.8 (6)
P1—C19—H19B	109.5	C37—C38—H38	120.6
H19A—C19—H19B	108.0	C33—C38—H38	120.6
C34—C33—C38	120.2 (6)	C13—C14—C15	118.4 (8)
C34—C33—P2	118.3 (4)	C13—C14—H14	120.8
C38—C33—P2	121.2 (5)	C15—C14—H14	120.8
C19—C20—P2	113.4 (4)	C36—C37—C38	120.1 (6)
C19—C20—H20A	108.9	C36—C37—H37	120.0
P2—C20—H20A	108.9	C38—C37—H37	120.0
C19—C20—H20B	108.9	C17—C18—C13	118.6 (8)
P2—C20—H20B	108.9	C17—C18—H18	120.7
H20A—C20—H20B	107.7	C13—C18—H18	120.7
C26—C21—C22	120.8 (6)	C16—C15—C14	120.5 (9)
C26—C21—P2	121.3 (5)	C16—C15—H15	119.8
C22—C21—P2	117.8 (5)	C14—C15—H15	119.8
C11—C12—C7	119.0 (7)	C36—C35—C34	119.4 (7)
C11—C12—H12	120.5	C36—C35—H35	120.3
C7—C12—H12	120.5	C34—C35—H35	120.3
C5—C6—C1	120.2 (7)	C15—C16—C17	121.4 (7)
C5—C6—H6	119.9	C15—C16—H16	119.3
C1—C6—H6	119.9	C17—C16—H16	119.3
C21—C26—C25	119.3 (7)	C35—C36—C37	121.0 (7)
C21—C26—H26	120.3	C35—C36—H36	119.5
C25—C26—H26	120.3	C37—C36—H36	119.5
			
C7—P1—C13—C14	−25.4 (6)	C20—P2—C21—C22	−86.0 (5)
C1—P1—C13—C14	−145.5 (5)	C8—C7—C12—C11	0.7 (9)
C19—P1—C13—C14	94.8 (6)	P1—C7—C12—C11	−170.0 (5)
C7—P1—C13—C18	159.2 (5)	C2—C1—C6—C5	0.4 (10)
C1—P1—C13—C18	39.1 (6)	P1—C1—C6—C5	175.0 (5)
C19—P1—C13—C18	−80.6 (5)	C22—C21—C26—C25	−1.4 (10)
C1—P1—C7—C8	−90.0 (5)	P2—C21—C26—C25	179.7 (5)
C13—P1—C7—C8	148.9 (5)	C28—C27—C32—C31	−2.0 (10)
C19—P1—C7—C8	30.7 (5)	P2—C27—C32—C31	177.8 (5)
C1—P1—C7—C12	80.8 (5)	C12—C7—C8—C9	−0.3 (10)
C13—P1—C7—C12	−40.3 (5)	P1—C7—C8—C9	170.5 (6)
C19—P1—C7—C12	−158.5 (5)	C29—C30—C31—C32	0.0 (12)
C33—P2—C27—C32	109.9 (5)	C27—C32—C31—C30	1.1 (11)
C21—P2—C27—C32	−130.9 (5)	C7—C8—C9—C10	−0.9 (12)
C20—P2—C27—C32	−11.3 (6)	C31—C30—C29—C28	−0.3 (13)
C33—P2—C27—C28	−70.3 (6)	C27—C28—C29—C30	−0.5 (12)
C21—P2—C27—C28	49.0 (6)	C6—C1—C2—C3	−1.8 (11)
C20—P2—C27—C28	168.5 (5)	P1—C1—C2—C3	−176.2 (6)
C7—P1—C1—C2	91.2 (6)	C1—C6—C5—C4	1.3 (11)
C13—P1—C1—C2	−146.4 (5)	C3—C4—C5—C6	−1.6 (12)
C19—P1—C1—C2	−29.1 (6)	C7—C12—C11—C10	0.0 (11)
C7—P1—C1—C6	−83.3 (5)	C21—C22—C23—C24	−0.9 (12)
C13—P1—C1—C6	39.2 (5)	C8—C9—C10—C11	1.6 (14)
C19—P1—C1—C6	156.4 (5)	C12—C11—C10—C9	−1.1 (13)
C32—C27—C28—C29	1.7 (10)	C5—C4—C3—C2	0.2 (13)
P2—C27—C28—C29	−178.2 (6)	C1—C2—C3—C4	1.5 (13)
C7—P1—C19—C20	61.5 (4)	C21—C26—C25—C24	−0.5 (11)
C1—P1—C19—C20	−179.4 (4)	C22—C23—C24—C25	−0.9 (13)
C13—P1—C19—C20	−60.1 (5)	C26—C25—C24—C23	1.6 (13)
C27—P2—C33—C34	74.2 (6)	C38—C33—C34—C35	−0.4 (10)
C21—P2—C33—C34	−44.2 (6)	P2—C33—C34—C35	−174.6 (6)
C20—P2—C33—C34	−165.6 (5)	C34—C33—C38—C37	1.0 (9)
C27—P2—C33—C38	−100.0 (5)	P2—C33—C38—C37	175.1 (5)
C21—P2—C33—C38	141.5 (5)	C18—C13—C14—C15	0.5 (10)
C20—P2—C33—C38	20.2 (6)	P1—C13—C14—C15	−174.7 (5)
P1—C19—C20—P2	136.1 (3)	C33—C38—C37—C36	0.0 (10)
C27—P2—C20—C19	−176.4 (4)	C16—C17—C18—C13	−0.5 (12)
C33—P2—C20—C19	62.5 (5)	C14—C13—C18—C17	−0.2 (10)
C21—P2—C20—C19	−58.2 (5)	P1—C13—C18—C17	175.1 (6)
C23—C22—C21—C26	2.1 (10)	C13—C14—C15—C16	0.0 (12)
C23—C22—C21—P2	−179.0 (6)	C33—C34—C35—C36	−1.2 (12)
C27—P2—C21—C26	−148.4 (5)	C14—C15—C16—C17	−0.7 (14)
C33—P2—C21—C26	−28.5 (6)	C18—C17—C16—C15	1.0 (14)
C20—P2—C21—C26	93.0 (5)	C34—C35—C36—C37	2.1 (13)
C27—P2—C21—C22	32.7 (6)	C38—C37—C36—C35	−1.6 (12)
C33—P2—C21—C22	152.6 (5)		

### Hydrogen-bond geometry (Å, °)

**Table:**

*D*—H···*A*	*D*—H	H···*A*	*D*···*A*	*D*—H···*A*
C30—H30···O5^i^	0.93	2.43	3.351 (10)	172
C20—H20B···O8^ii^	0.97	2.38	3.338 (11)	170
C19—H19B···O4	0.97	2.44	3.397 (8)	171
C19—H19A···O7	0.97	2.30	3.135 (8)	144

Symmetry codes: (i) *x*−1/2, −*y*+3/2, *z*+1/2; (ii) −*x*+3/2, *y*+1/2, −*z*+1/2.
